# Papillary Thyroid Cancer Affecting Multiple Family Members: A Case Report and Literature Review of Familial Nonmedullary Thyroid Cancer

**DOI:** 10.1155/2021/3472000

**Published:** 2021-10-15

**Authors:** Khaled Ahmed Baagar, Buthina Ibrahim Alowainati

**Affiliations:** Endocrine Department, Hamad Medical Corporation, Doha, P.O. Box 3050, Qatar

## Abstract

Familial nonmedullary thyroid cancer (FNMTC) represents 5–10% of NMTC cases. Many controversies are associated with the FNMTC, namely, the minimum required number of affected family members to define the condition, aggressiveness, prognosis, and treatment and screening recommendations. Moreover, the genetic basis of the FNMTC has not yet been identified. We report a family diagnosed with FNMTC and present a comprehensive literature review of the condition. The index case was a 26-year-old male who was diagnosed with locally advanced papillary thyroid cancer (PTC). Then, his family members became worried and asked for a neck ultrasound. Four of his six siblings, in addition to his father, were diagnosed with PTC. In addition, two of his cousins were diagnosed. The patient underwent total thyroidectomy with bilateral neck dissection, and he received 2 doses of radioactive iodine (100 mCi each). Furthermore, one of his siblings required a second surgery with repeated radioactive iodine therapy. The index case genetic screening and whole-exome sequencing did not show any abnormalities. Future genetic and clinical research should focus on kindred with 3 or more affected individuals for better identification of the FNMTC susceptibility genes and to better guide management and screening recommendations.

## 1. Introduction

Papillary thyroid cancer (PTC) is the most common primary thyroid malignancy and accounts for more than 80% of thyroid cancers [1]. Most PTC cases are sporadic; however, 5–10% are familial [2, 3], and like the sporadic form, females are 2–3 times more affected than males [4–6]. Familial PTC (FPTC) is the most common type of familial nonmedullary thyroid cancer (FNMTC) [7], which is identified, in most studies, when two or more first-degree relatives are diagnosed with the condition in the absence of a history of familial syndromes or risk factors that are commonly associated with NMTC [8–11].

We report a family which was diagnosed with FPTC after the index case (i.e., the first case had been diagnosed in the family) had been diagnosed with a locally advanced PTC. In addition, in this report, we reviewed the topic of FNMTC with its associated controversies in definition, aggressiveness in comparison to sporadic NMTC (SNMTC), latest information about the genetic background, prognosis, screening and management recommendations, and future research requirements to improve our knowledge about the condition.

## 2. Case Presentation

In February 2015, a 26-year-old male presented to the emergency department with generalized muscle weakness for 1 day. On physical examination, he had normal vital signs except for a pulse rate of 105/minute, a muscle power of 3/5 throughout his extremities, and a multinodular goiter with bilateral cervical lymphadenopathy. His laboratory workup was significant for potassium: 1.8 mmol/L (normal: 3.6–5), TSH: <0.01 mIU/L (normal: 0.45–4.5), FT4: 22.2 pmol/L (normal: 9–20), and FT3: 10 pmol/L (normal: 3.4–6). His muscle power improved with potassium replacement. A thyroid uptake scan showed increased uptake with a cold thyroid nodule. Therefore, carbimazole and propranolol were prescribed.

Neck ultrasound and magnetic resonance imaging showed 1.6 cm and 1.2 cm nodules with calcifications in the right thyroid lobe, with evidence of bilateral lymph node metastases and bilateral extranodal soft tissue metastases. CT chest to assess mediastinal lymphadenopathy or lung metastases was unremarkable. Fine-needle aspiration biopsy (FNAB) from a lymph node metastasis in the neck showed metastatic PTC. Then, FNAB from the right thyroid nodules was performed, and it showed PTC.

The patient underwent total thyroidectomy and bilateral neck dissection. Two foci (2 cm and 1 cm) of classical PTC were found in the right thyroid lobe with lymph node involvement and extranodal metastases. Thereafter, I^131^ 100 mci was given, and the postablation scan showed a remnant in the left thyroid bed (TNM staging: T4aN1bM0 tumor).

**Figure 1:**
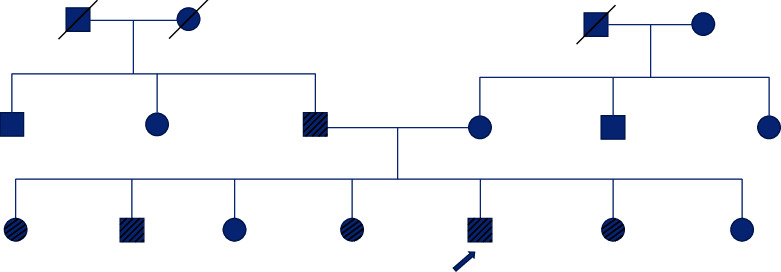
Family pedigree. Squares represent male family members; circles represent female family members; multiple-slashed symbols represent affected members; single-slashed symbols represent deceased members. The arrow points to the proband.

The patient's family was worried about themselves and asked for a thyroid evaluation. The patient's brother, in addition to 3 of his 5 sisters, was found to have PTC (see details in Table 1). The remaining 2 sisters (aged 22 and 29 years) and the mother (aged 52 years) underwent a normal initial thyroid ultrasound scan, and then the yearly ultrasound surveillance was unremarkable. The father (aged 55 years) was found to have a thyroid nodule; however, he was reluctant to have FNAB until January 2018 at another institution where he was diagnosed with thyroid cancer, but we do not have any data about his follow-up there (see family pedigree in Figure 1).

There were no other cancers or clinical features indicating a familial syndrome in the family. The patient's genetic screening was negative for pathogenic variants of the PARP4, APC, DICER1, RET, SDHB, and SDHD genes. In addition, the patient underwent whole-exome sequencing, which was unremarkable.

After more than 6 years of follow-up of the index case and his family, two of them, including himself, had a recurrence and required further treatment (see Table 1). In addition, he reported to us that 2 of his paternal cousins, who were not siblings, had developed thyroid cancer.

## 3. Discussion

In 1955, Robinson and Orr presented the first evidence of the familial clustering of NMTC, where they reported PTC in 2 monozygotic twins [12].

Many controversies are related to the FNMTC, starting from its definition to clinical behavior, outcomes, management recommendations, and screening of the patients' family members. This controversy is mainly because of the small number of studies of FNMTC, mostly retrospective, and their relatively small number of subjects because of the low disease prevalence. In addition, the follow-up duration in these studies was short relative to the nature of the disease.

There is no consensus on whether two [2, 9, 13, 14] or three [15–18] first-degree relatives of a family are the minimum required number to define FNMTC, as SNMTC is very common and it can affect more than one individual in a family. Charkes [5] found that in families with 2 affected individuals, 62–69% of the cases are sporadic, while in families with ≥3 affected members, less than 6% of the cases are sporadic. In one study, it was found that 4.6% of at-risk individuals had thyroid cancer in families with 2 affected members, while 22.7% of at-risk individuals were affected in the families with ≥3 affected members [19].

Despite the recommendation to consider mainly families with ≥ 3 affected members for clinical and genetic analyses [5], most of the reports are mainly based on families with 2 affected cases. The family we described in this report, with 6 affected first-degree relatives, has very strong evidence of FNMTC.

### 3.1. Histopathological Features

From a histological viewpoint, there is no specific feature that distinguishes FNMTC from SNMTC [20], except in one report which showed that patients in the FNMTC group had more significantly classic variant PTC than those in the sporadic PTC group (84% vs. 63.3%; *P* < 0.001) [7]. We found that all the affected individuals in the reported family had classic variant PTC, except one who had a follicular variant PTC.

### 3.2. Clinical Aggressiveness at Presentation

It is the most controversial part, as some studies have shown that FNMTC demonstrates a more aggressive presentation than SNMTC, such as earlier onset, bilateral and multifocal tumors, extrathyroidal invasion, and lymph node metastasis [9, 14, 21–28].

Recently, El Lakis et al. reported that FNMTC patients were diagnosed at an earlier age (62% vs. 49.5%; *P*=0.041), with a higher rate at the T1 stage (75% vs. 57.8%; *P*=0.01) and a higher rate of lymph node involvement (*P*=0.002) [7].In addition, Sezer et al. found that both FPTC and sporadic PTC (SPTC) groups had no difference in age at presentation or tumor size; however, the familial group was found to have more aggressive features, such as higher rates of multifocal tumors (*P*=0.011), bilateral involvement (*P*=0.004), extrathyroid extension (*P*=0.040), and lymph node metastasis (*P*=0.013) [29]. Furthermore, Kim YS et al. reported more multifocality (*P*=0.020), benign thyroid nodules (*P*=0.015), and lateral neck lymph node involvement (*P*=0.021) in the FNMTC group [10].

The reported family had 6 affected members, with 2 of them (the index case and sibling 3) showing the most aggressive features such as multifocality, capsular invasion, extrathyroid extension, vascular/lymphatic invasion, and lymph node involvement and sibling 3 showing bilateral disease.

Even those with familial papillary microcarcinoma, when compared to their sporadic counterparts, had more multifocal tumors (71%), more vascular invasion (43%), and a higher rate of lymph node involvement (57%) [30]. In the reported family, only sibling 4 had a microscopic PTC with no aggressive features.

On the other hand, a good number of studies did not show any difference in clinical aggressiveness at presentation between FNMTC and SNMTC patients [1, 6, 18, 31–33].

Most studies, whether they showed or did not show that FNMTC was more aggressive than SNMTC, did not show a difference in the rate of distant metastasis. However, Sezer et al. reported a higher rate in the familial group (*P* ≤ 0.0001) [29], and McDonald et al. [34] found that the FNMTC group had more distant metastasis (8.8%) than the SNMTC group (3.1%) (*P*=0.003), with more aggregation of the cases in some families with ≥3 affected members.

Some studies have tried to investigate whether there is any difference in the clinical behavior of the FNMTCs when only 2 first-degree relatives are affected and when ≥3 first-degree relatives are affected. In the study performed by Zhang et al. [9], the FNMTC subgroup with 2 affected members had more significant multifocal tumors than the SNMTC group. The FNMTC subgroup with ≥3 affected members had more significant thyroid nodular goiter and bilateral and multifocal tumors with extrathyroid extension and lateral lymph node metastasis than both the SNMTC group and the FNMTC subgroup with 2 affected members. On the other hand, Pinto et al. [13] showed that the FNMTC subgroup with ≥3 affected members had no difference from the SNMTC group regarding the clinicopathological features or DNA ploidy, with only a nonsignificant tendency for extrathyroid invasion in that subgroup. In addition, McDonald et al. [34] showed no difference in almost the same surgical pathology features between the SNMTC, 2-member FNMTC, and ≥3-member FNMTC groups.

In more recent studies, El Lakis et al. found no difference between the FNMTC subgroup of 2 affected members and either the SNMTC group or the FNMTC subgroup of ≥3 affected members; however, the latter group presented at a younger age (*P*=0.04) and had more lymph node metastasis (*P*=0.009) than the SNMTC group [7]. On the other hand, Sezer et al. showed that the 2-member FPTC group, in comparison to the SPTC group, had more significant bilaterality, multifocality, extrathyroid extension, and distant metastasis [29]. The ≥3-member FPTC group, in comparison to the SPTC group, had more distant metastasis and less extrathyroid extension [29]. The comparison of the 2-member FPTC group and the ≥3-member FPTC group showed that the former had more vascular invasion and extrathyroid extension and lower rates of bilaterality, lymphatic invasion, and distant metastasis [29].

A meta-analysis of 6 retrospective studies, with a relatively small number of subjects, comparing the clinicopathological features between the first and second generations in families with FNMTC found that second-generation patients tend to be younger, male, and have a higher risk of lymph node metastasis. However, there was no difference in tumor size, multifocality, or extrathyroid invasion [8].

One study tried to compare 2 subtypes of FNMTC, namely, parent-offspring type and sibling type versus each other and versus SNMTC [27]. When compared to SNMTC, the sibling FNMTC group had more females, smaller tumor size, and more combined chronic thyroiditis; however, there was no difference in age at the time of diagnosis, lymph node metastasis, extrathyroid invasion, multicentricity, or TNM staging. The parent-offspring FNMTC group had more multicentricity than the SNMTC group. The sibling FNMTC group had only more females than the parent-offspring FNMTC group. Another study found that the sibling FNMTC group was associated with more Hashimoto's thyroiditis and extrathyroid extension than the parent-offspring FNMTC group [14]. Furthermore, in comparison to the SNMTC group, the sibling FNMTC group had more bilateral and multicentric tumors, while the parent-offspring FNMTC group showed more Hashimoto's thyroiditis, central lymph node metastasis, and T3 tumors [14].

Another type of comparison was performed by Rosario et al. [35] between index cases and their affected siblings who were screened by ultrasound. The index cases were found to have an older average age at the time of diagnosis, larger tumors, more lymph node metastases, and more extrathyroid invasion. In addition, McDonald et al. [34] reported that the index cases had a more aggressive disease because the other family members sought screening and they were diagnosed at an earlier stage of the disease. Recent studies reported that the index cases had larger tumor sizes and more lymph node metastases, and because of that, they required more aggressive surgeries and radioactive iodine therapy [7, 19]. The index case in our report had the most aggressive disease (T4aN1bM0) in comparison to his siblings, and he required more extensive initial surgery in addition to 2 radioactive iodine ablation therapy doses of 100 mCi each.

### 3.3. Prognosis (Recurrence, Disease-Free Survival, and Survival)

In general, PTC has good survival rate with only 6% malignancy-related mortality at a median follow-up of 16 years in patients with nonmetastatic disease at presentation [36]. However, the controversy continues with the prognosis of FNMTC, where some studies showed that it has worse outcomes than SNMTC, while others did not show a difference.

The rate of tumor recurrence in FNMTC was higher than that in SNMTC in a number of publications [9, 22, 28, 34], while it did not differ in others [6, 10, 13, 14, 18, 31, 33].

After more than 6 years of follow-up of the reported family, the 2 members who had more aggressive tumors at presentation developed recurrent disease and required more aggressive surgeries and repeated radioactive iodine ablation therapy.

Only a few studies have assessed the disease-free survival (i.e., time to tumor recurrence), and it was shorter in FNMTC [9, 22, 24, 27, 28].

Regarding mortality in association with FNMTC, most of the studies showed no difference from SNMTC [1, 7, 9, 10, 13, 14, 22, 24, 31, 33, 37], except in the study carried out by McDonald et al. [34], where the FNMTC group had more significant mortality (3.3% of 91 cases) than the SNMTC group (0.4% of 521 cases) (*P*=0.01).

Some studies tried to compare the different subtypes of FNMTC prognostically. For example, in one study [9], the disease-free survival was shorter in families with ≥3 affected members than in families with only 2 victims (*P*=0.045), while in other studies [18, 27, 33], there was no difference. In addition, there was no difference between these two FNMTC subgroups in persistence or recurrence of the disease or in mortality [9]. Furthermore, there was no difference in the recurrence rate between sibling and parent-offspring subtypes of FNMTC [14, 27] or in a meta-analysis [8] comparing first-generation versus second-generation patients in families with FNMTC.

### 3.4. Pathogenesis and Genetic Background

Sometimes FNMTC is referred to as syndromic and nonsyndromic [20]. The syndromic group is not consistent with the true definition of the FNMTC, where FNMTC should not be a part of a familial syndrome, e.g., familial adenomatous polyposis, Werner syndrome, Cowden syndrome, Pendred syndrome, and Carney complex. Nonsyndromic FNMTC can be subdivided into different forms, namely, pure familial PTC with or without oxyphilia, familial PTC with papillary renal cell carcinoma, and familial PTC with multinodular goiter [20].

The genetic background of the nonsyndromic FNMTC is still unknown. However, based on the studied kindred, it is thought to be polygenic and autosomal dominant with incomplete penetrance and variable expression [20, 38].

In addition, FNMTC demonstrated the phenomenon of genetic anticipation, where with successive generations, the disease is diagnosed at an earlier age with a more aggressive presentation [14, 16, 39].

The genetic testing of the proband in the reported family did not show any genetic abnormality linked to his phenotype.

Research to identify FNMTC susceptibility genes is still ongoing. There are different potential genetic loci and genes that have been reported, such as multinodular goiter 1 (MNG1; 14q32) [40], thyroid carcinoma with oxyphilia (TCO; 19p13.2) [41], fPTC/papillary renal neoplasia (PRN; 1q21) [42], NMTC1 (2q21) [43], familial thyroid epithelial neoplasia (FTEN; 8p23.1–p22) [44], telomere-telomerase complex [45], TITF-1/NKX2(which encodes thyroid transcription factor-1) [46], SRGAP1 (Slit- Robo Rho GTPase activating protein 1) genes [47], hyaluronan binding protein 2 (HABP2) [48], FOXE1 [49], SRRM2 (serine/arginine repetitive matrix 2 gene) [50], C14orf93 (RTFC) [51], mitogen-activated protein kinase kinase 5 (MAP2K5) [52], CHEK2 and ATM genes [53], ANXA3, NTN4, SERPINA1, FKBP10, PLEKHG5, P2RX5, SAPCD1 [54], and protection of telomeres 1 [POT1] gene [55].

The reason that, until now, no definite FNMTC susceptibility genes have been identified may be related to the inclusion of families with only 2 affected members in the genetic studies, while NMTC is not an uncommon condition in the general population. In addition, other modes of inheritance should be studied, such as the possible role of epigenetics [56].

### 3.5. Screening and Management Recommendations

There are no randomized controlled trials to guide the screening and management recommendations of FNMTC, and the available evidence only came from observational studies and expert opinion.

We need to take into consideration that recommending a screening program for any cancer condition requires the following[19]:

1. To be directed to at-risk individuals
2. To be able to discover the disease early in its course
3. That early detection of the disease impacts the outcome
4. To be cost-effective

Currently, the best available screening method is the ultrasound neck [2, 14, 19, 57], as there is no genetic test to definitely diagnose FNMTC and, for example, guide prophylactic thyroidectomy similar to familial medullary thyroid cancer [2].In addition, physical examination alone can miss many cases with nodular thyroid disease [19, 35]. Klubo-Gwiezdzinska et al. reported screening of 109 at-risk individuals, of which 55 (50.5%) had thyroid nodules detected by ultrasound; however, only 7 cases (7/55 = 12.7%) were palpable on physical examination.

Screening can detect FNMTC cases at an earlier stage of the disease; however, there is no consensus on at what age ultrasound screening should start and how frequently it should be performed. The youngest diagnosed patient with FNMTC was aged 8 years [13]. Some authors [14] recommended starting ultrasound screening from the age of 18 years and performing ultrasound screening yearly afterward, while others advised screening from the age of 10 years [2].

Most of the published management recommendations endorse more aggressive treatment, including total thyroidectomy, central lymph node dissection, radioactive iodine ablation therapy, and suppressive thyroid hormone treatment, in almost all patients with FNMTC [9, 10, 14, 58].In addition, the treatment of FNMTC in families with 3 or more affected members should be more aggressive, as they usually have a more hostile disease [7].

Capezzone et al. suggested that management recommendations for FNMTC should depend on controlled studies with comparable groups of FNMTC cases and SNMTC cases in their baseline characteristics [11].Furthermore, as the latest recommendations for less aggressive management of SNMTC were not assessed specifically in those with FNMTC, the authors suggested total thyroidectomy and radioactive iodine ablation therapy, especially for those belonging to families with 3 or more affected members [11].

Mazeh and Sippel [57] suggested an algorithm for screening and management of FNMTC where treatment decisions took into consideration the number of affected family members and the degree of aggressiveness of the disease within the family. They suggested performing a neck ultrasound screening on all family members starting from the age of 20 years or 10 years younger than the youngest affected family member. If there is no nodule, periodic ultrasound should be performed. If the person has benign nodules based on FNAB, prophylactic thyroidectomy could be performed if the family has an aggressive disease or has 3 or more affected members. They justified this recommendation based on families with FNMTC being more likely to have benign and malignant thyroid disease, which lowers the chance to detect cancer. If cancer was identified by FNAB, they recommend total thyroidectomy, routine central neck dissection in almost all patients, radioactive iodine ablation therapy if the family is known to have an aggressive disease or has 3 or more affected members, and thyroid hormone suppressive therapy in all patients.

Recently, Klubo-Gwiezdzinska et al. [19] authored a prospective study using neck ultrasound screening and reported a cancer detection rate of 4.6% in families with 2 members affected, which is similar to the general population (4.5%) [1, 59]. However, in families with ≥3 members affected, the detection rate was 22.7% (*P*=0.01).In addition, thyroid nodules were documented in 72.2% of the parents of index cases. Furthermore, the FNMTC detected in the screened cases was less aggressive than the index cases and required less aggressive surgery (hemithyroidectomy 23.5% versus 0%; *P*=0.002) and a lower rate of radioactive iodine ablation therapy (*P* ≤ 0.001), with a better response to initial treatment than the index cases (*P*=0.045). Based on their data, the authors recommended active screening using neck ultrasound only for families with 3 or more affected first-degree relatives. The screening of first-degree relatives should include all generations relative to the index case and involve those at least aged 20 years or 10 years younger than the youngest diagnosed case with NMTC. Neck ultrasound should be performed every 2–3 years.

The latest American Thyroid Association (2015) guidelines did not recommend or discourage using neck ultrasound for FNMTC screening [60]. However, in view of the recent accumulating evidence, families with ≥3 affected first-degree relatives should be considered eligible for ultrasound screening. In addition, to avoid overdiagnosis and overtreatment of clinically insignificant thyroid cancer cases in the population, it is better not to implement a screening program for families with only 2 affected members.

In the reported family, we performed neck ultrasound screening for siblings and parents. In addition, we advised them to start screening their children when they reach the age of 13 years (10 years less than the youngest diagnosed member in the family) and to repeat it yearly for the unaffected family members. All the family members diagnosed after the index case underwent total thyroidectomy with central lymph node dissection, including sibling 4 with microscopic PTC.

### 3.6. Future Research

Future research should focus on the identification of a solid molecular basis of FNMTC, as this is the best way to diagnose true familial cases. This will help to conduct further studies to develop evidence-based recommendations for patient management and follow-up and for family screening protocols.

Limiting the definition of FNMTC to include only kindred with 3 or more affected members may help in better characterization of the susceptibility genes, and this, in turn, helps to endorse more solid screening and management recommendations.

Because of the low prevalence of FNMTC, research and clinical centers in different countries should collaborate to have enough patients to obtain meaningful results.

## 4. Conclusions

FNMTC has many controversies in relation to its definition, clinicopathological aggressiveness, prognosis, treatment, and screening of at-risk family members. Different genetic mutations have been investigated; however, none of them was confirmed to be a source of the condition. Based on the available evidence, it is advisable to implement ultrasound neck screening only in families with 3 or more affected members and to be done yearly starting at age 10 years earlier than the youngest affected family member. In addition, the diagnosed cases in those families should have a lower threshold for a more aggressive management decision.

The multidisciplinary team of endocrinologists, surgeons, and genetics specialists at our institution recommended the same for the reported family.

FNMTC is an area that needs to be addressed in future research to resolve its ambiguity and controversial issues and to have screening and management recommendations based on high-quality evidence.

## Figures and Tables

**Table 1:** Characteristics of the diagnosed thyroid cancer in the family members.

	Index case	Sibling 1	Sibling 2	Sibling 3	Sibling 4	Father
Age at diagnosis (years)	26	30	23	29	33	58
Gender	Male	Male	Female	Female	Female	Male
Nodular goiter (±)	+	−	−	+	+	
Tumor diameter (cm)	2.0	1.0	2.2	2.6	0.12	
Histological variant of PTC	Classic	Classic	Classic	Classic	Follicular	
Laterality (unilateral/bilateral)	Unilateral	Unilateral	Unilateral	Bilateral	Unilateral	
Focality (unifocal/multifocal)	Multifocal	Unifocal	Unifocal	Multifocal	Unifocal	
Capsule invasion (±)	+	+	−	+	−	
Extrathyroid extension (±)	+	−	−	+	−	
Vascular/lymphatic invasion	+	−	−	+	−	
Hashimoto's thyroiditis (±)	−	−	+	−	−	
Central LN involvement (±)	+	−	−	+	−	
Lateral LN involvement (±)	+	Not sampled	Not sampled	Not sampled	Not sampled	
Distant metastasis (±)	−	−	−	−	−	
pTNM classification	T4aN1bM0	T1aN0M0	T2N0M0	T3bN1aM0	T1aN0Mx	
Surgical procedures	Total thyroidectomy with bilateral neck dissection	Total thyroidectomy with central LN dissection	Total thyroidectomy with central LN dissection	Total thyroidectomy with central LN dissection	Total thyroidectomy with central LN dissection	
RAI ablation and dose	+, 100 mCi	+, 100 mCi	−	+, 30 mCi	−	
Persistence/recurrence of the disease (±)	+	−	−	+	−	
Repeated treatment (surgery and/or RAI and dose)	In February 2017, he received a 2nd dose of RAI (100 mCi) for local recurrence in the thyroid bed	−	−	In April 2019, surgery for thyroid recurrence with bilateral neck dissection and RAI (100 mCi)	−	
Death from the disease (±)	−	−	−	−	−	−
Duration of follow-up	Since April 2015 (75 months)	Since July 2015 (72 months)	Since November 2015 (68 months)	Since May 2016 (62 months)	Since August 2015 (71 months)	Since January 2018 (42 months)

PTC: papillary thyroid cancer, LN: lymph node, and RAI: radioactive iodine. *Note.* There were no data about the father's tumor, as he had the management and follow-up data outside our institution.

## Data Availability

The data used to support the findings of this study are included within the article.
